# Supplementary material for: Van der Waals interaction affects wrinkle formation in two-dimensional materials
Source: Proc Natl Acad Sci U S A. 2021 Mar 31;118(14):e2025870118. doi: 10.1073/pnas.2025870118 (PMC8040643; doi:10.1073/pnas.2025870118)
Supplement: Supplementary File [file pnas.2025870118.sapp.pdf]

PNAS
www.pnas.org

Supplementary Information for

van der Waals interaction affects wrinkle formation in two-dimensional materials

Pablo Ares[a,b,*], Yi Bo Wang[a,b], Colin R. Woods[a,b], James Dougherty[a,b], Laura Fumagalli[a,b], Francisco Guinea[c,d,e], Benny Davidovitch[f,†], Kostya S. Novoselov[a,b,g,h,‡]

[a]Department of Physics & Astronomy, University of Manchester, M13 9PL Manchester, UK
[b]National Graphene Institute, University of Manchester, Manchester, M13 9PL Manchester, UK
[c]IMDEA (Instituto Madrileño de Estudios Avanzados) Nanoscience, 28049 Madrid, Spain
[d]Donostia International Physics Center, 20018 San Sebastián, Spain
[e]Ikerbasque, Basque Foundation for Science, 48009 Bilbao, Spain
[f]Department of Physics, University of Massachusetts Amherst, Amherst, MA 01003, USA
[g]Centre for Advanced 2D Materials, National University of Singapore, Singapore 117546, Singapore
[h]Chongqing 2D Materials Institute, Chongqing 400714, People's Republic of China

*Email: pableras.ares@gmail.com
†Email: bdavidov@umass.edu
‡Email: kostya@nus.edu.sg

This PDF file includes:

Figures S1 to S4
Supplementary text
Table S1
SI References

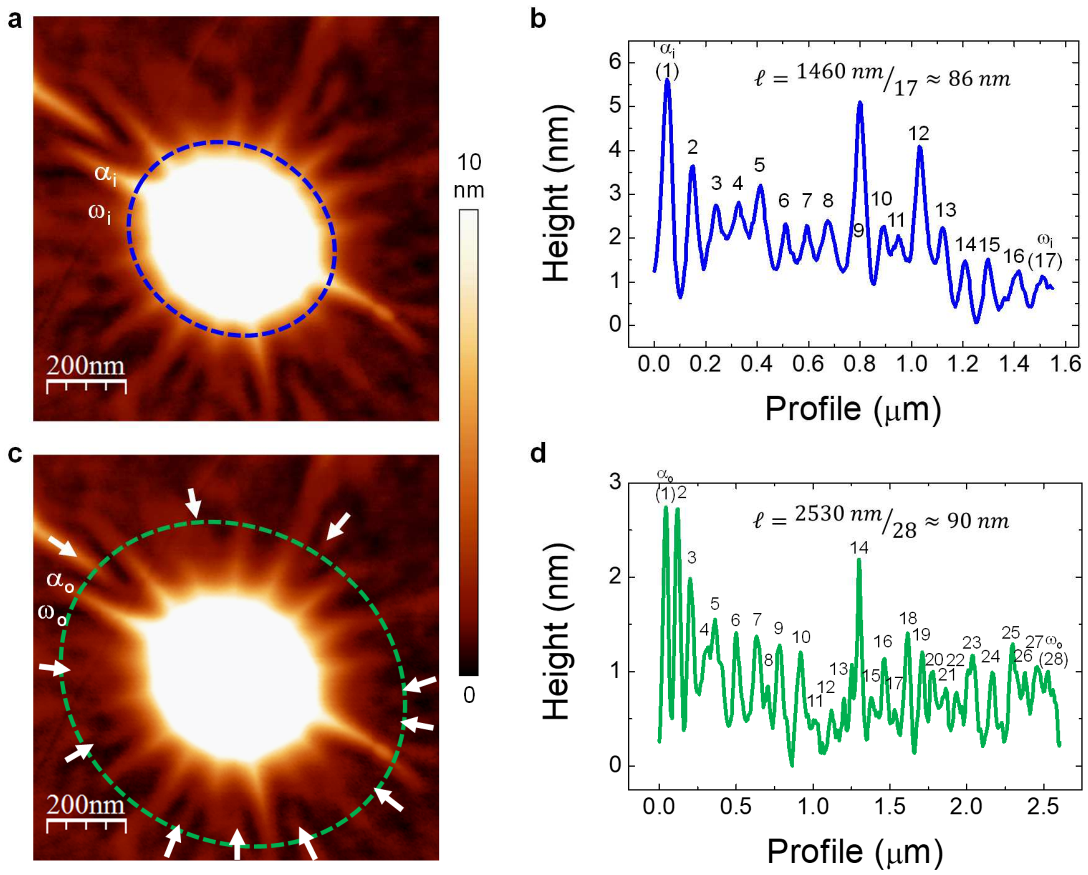

Fig. S1. Determination of the wrinkle number m(r) for wavelength calculation. Panels reproduced from Fig.1 in the main text for clarity. **a**, and **c**, Topographic image of wrinkles around a bubble in 2L hBN. Height scale: 10 nm. **b**, and **d**, Height profiles along the dashed lines in **a**, and **c** respectively. The letters α and ω refer to the first and last wrinkles in the profiles. Each wrinkle has been numbered for clarity.

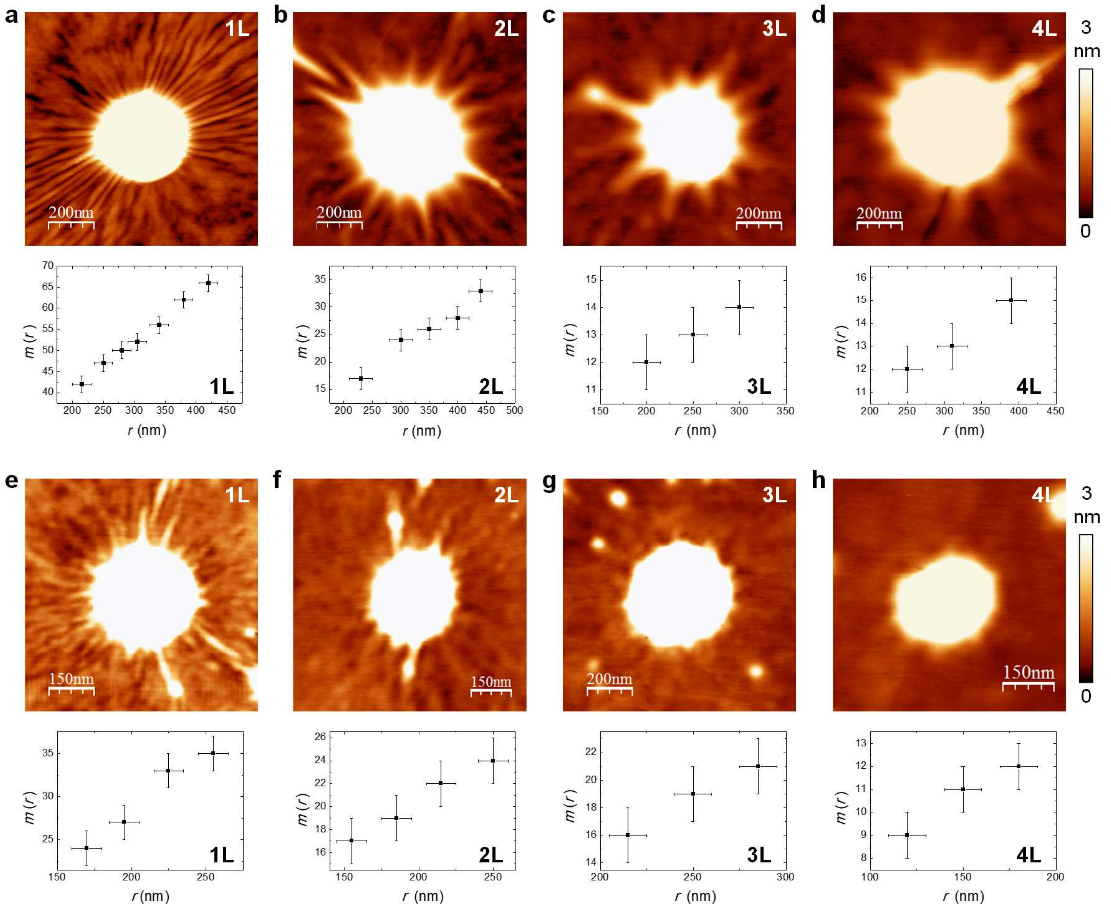

Fig. S2. Evolution of the wrinkle number *m(r)* with radial distance *r*. a-d, hBN and **e-h**, MoS2 topographic images (top rows) of bubbles with wrinkles for different layer thicknesses and corresponding wrinkle number *vs.* radial distance (bottom rows). In all the cases, the wrinkle number increases with the radial distance. Height scale: 3 nm.

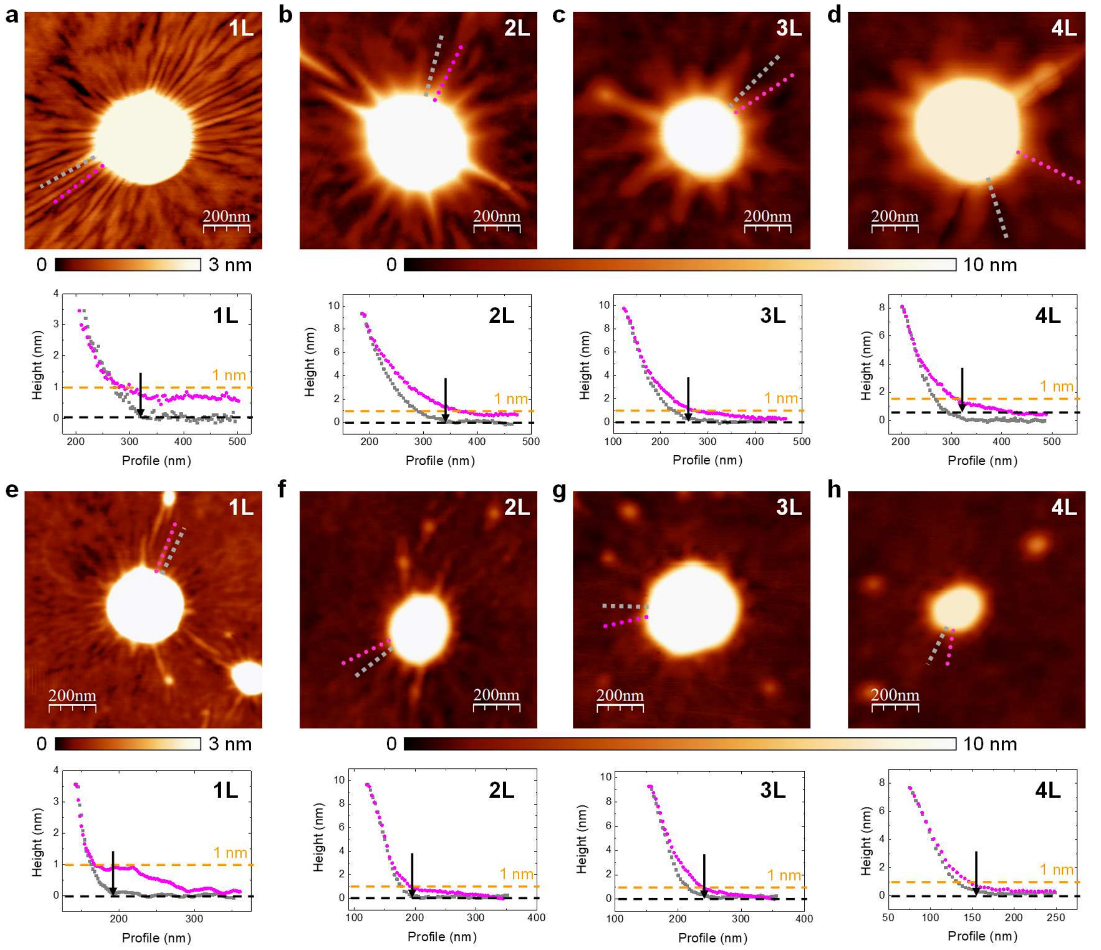

Fig. S3. Topographical images (top rows) and corresponding representative profiles (bottom rows) of wrinkles. a-d, hBN and **e-h**, MoS2 wrinkles in layers of different thicknesses. Height scales: 3 nm for 1L and 10 nm for the rest of the cases. The gray squared-dotted lines correspond to the substrate whereas the pink circled-dotted lines to wrinkles. The arrows in the profiles indicate when the bubble reaches the substrate level in the areas without wrinkles. In all the cases, the height of the wrinkles decays exponentially, such that most of the wrinkled zone is nearly flat in the radial direction and smaller than 1 nm in the majority of its length.

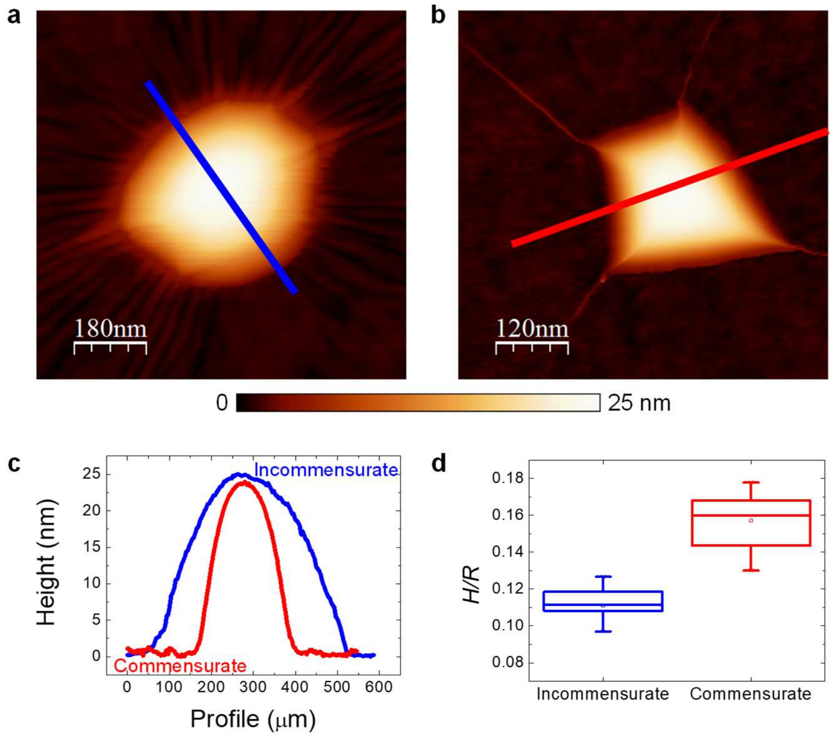

Fig. S4. Comparative of aspect ratio in bubbles present in incommensurate and commensurate states. a, Representative bubble in incommensurate and **b**, commensurate states. Height scale: 25 nm. **c**, Profiles along the lines in **a** and **b** showing a significant different aspect ratio for the two bubbles. **d**, Statistical analysis of the aspect ratio for the incommensurate and commensurate states.

Table S1. Elastic constants used for hBN and MoS2.

	**hBN**	**MoS2**
**λ (eVÅ^{-2})**	3.68 (1)	2.83 (2)
**μ (eVÅ^{-2})**	7.80 (1)	3.135 (2)
***d* (Å)**	3.34	6.5
***B* (eV)**	0.85 (3)	10 (4)

Substrate-induced stiffness of a membrane on a supporting substrate

The effective stiffness $K_{eff}(r)$ can originate from multiple contributions (5):

$$K_{eff}(r) = K_{sub} + K_{tens}(r) + K_{curv}(r) \qquad (1)$$

Where $K_{eff}(r)$ ([force]/[length]^3, alternatively [energy]/[length]^4) acts to suppress the defection (amplitude) of wrinkles from the rest (naturally planar) state of the membrane. Generally, the effective stiffness, $K_{eff}(r)$, comprises three types of restoring (amplitude-suppressing) forces:

i. A substrate-induced stiffness, K_{sub} , attributed to an energetic cost, $K_{sub}A^2$, emanates from the normal force exerted on the membrane by a supporting substrate, $K_{sub} = V_{vdW}''(h_0)$. For polymer sheets, such a normal force reflects the energetic cost required to deform the substrate (*e.g.* Young's modulus of a compliant solid substrate (6), or the *g.p.e*. of a liquid bath). In our study, we can consider the supporting substrate as infinitely rigid and hence follow Zhang-Witten (7), assuming that the only normal force exerted by the substrate on the membrane originates from the change in the vdW energy. Namely, $K_{sub} \cdot A = V_{vdW}''(h_0) \cdot A$ is the force required to bring a unit area of the membrane to a distance $h = h_0 + A$ from the substrate (where $h = h_0$ is the minimal-energy distance of a flat membrane from the substrate). Hence, $K_{sub} = V_{vdW}''(h_0)$.

ii. A tension-induced stiffness, $K_{tens}(r)$, is attributed to an energetic cost, $K_{tens}A^2 \propto \sigma_{rr} A'(r)^2$, of radial variation of the wrinkle amplitude, $A(r)$, in the presence of radial tension $\sigma_{rr} \sim \Gamma^{1/2}Y^{1/2}$, *along* wrinkles (8). Since the radial variation of the wrinkle amplitude occurs over a scale $\propto R$, we may estimate, $K_{tens} \sim \Gamma^{1/2}Y^{1/2}/R^2$.

iii. A curvature-induced stiffness, $K_{curv}(r) \sim Y\,[z_0''(r)]^2$, is attributed to an energetic cost of the strain associated with deforming a curved "envelope" shape, $z_0(r)$, with radial curvature $\sim z_0''(r)$, due to the Gaussian curvature induced by superimposing on it azimuthal undulations (5).

Our experimental observations enable us to rule out the relevance of $K_{curv}(r)$ and $K_{tens}(r)$. First, observation *(c)* in the main text (the non-oscillatory component of the wrinkles decays exponentially, such that most of the wrinkled zone is nearly flat in the radial direction, and highly curved in the azimuthal direction) implies that, while $K_{curv}(r)$ may be significant very close to the bubble, it decays exponentially rapidly, and does not affect the membrane in the vast part of the wrinkled zone. Second, observation *(e)* (for a given type and number of layers of the top membrane, the average wavelength ℓ_0 does not depend on the size (radius or height) of bubbles) implies that $K_{tens}(r)$ is also negligible, otherwise the average wrinkle wavelength would exhibit a strong dependence on the bubble's radius ($\ell_0 \propto \sqrt{R}$). Finally, observation *(b)* (the wrinkle number $m(r)$ increases with radial distance *r*) indicates a spatially-uniform wavelength, consistently with $K_{eff} \approx K_{sub}$.

A straightforward relation $K_{eff} \sim \Gamma\, d_{min}^{-2}$ could be used to estimate a characteristic width of the vdW potential well. This expression leads to length scales of the order of 1 nm for the commensurate case, and 10 nm for the incommensurate one. These estimates, which do not seem realistic, will be significantly reduced if one considers, as seems likely, that the very amplitude of the wrinkles and the thermal fluctuations of the membranes at room temperature smooth the vdW potential.

Bending rigidity of stacks of weakly coupled layers

The bending rigidity of isotropic, or almost isotropic, elastic slabs is proportional to the in-plane bulk modulus, and it increases as the cube of the thickness of the slabs (9). A stack of layers coupled by the van der Waals interaction has highly anisotropic elastic constants, and a different response to bending.
The coupling between neighboring layers, at long wavelengths, can be described by three force constants, which determine i) the energy required for a change in the interlayer distance, that is, the breathing mode, ii) the energy required for a lateral displacement of one layer with respect the other, the shear mode, and iii) the coupling between a compression within the layer and a change in the interlayer distance, which determines the out of plane Poisson ratio.

The coupling between in plane deformations and bending in a slab arises from the shear deformation which arises when the slab is bent. Hence, it vanishes when the layers can freely slide one over the other. Neglecting the third coupling mentioned above, the elastic energy of a slab of N layers can be written as (10):

$$E = \sum_i \int d^2\vec{r} \left\{\frac{\lambda}{2}\left(\partial_x u_x^i + \partial_y u_y^i\right)^2 + \mu\left[(\partial_x u_x^i)^2 + \left(\partial_y u_y^i\right)^2 + \frac{1}{2}\left(\partial_x u_y^i + \partial_y u_x^i\right)^2\right]\right\} +$$
$$\frac{\Gamma_b}{2}\sum_i \int d^2\vec{r}_i \left(\frac{u_z^i - u_z^{i+1}}{d}\right)^2 + \frac{\Gamma_s}{2}\sum_i \int d^2\vec{r}_i \left[\left(\frac{-u_x^i + u_x^{i+1}}{d} + \frac{\partial_x u_z^i + \partial_x u_z^{i+1}}{2}\right)^2 + \left(\frac{u_y^i - u_y^{i+1}}{d} + \frac{\partial_y u_z^i + \partial_y u_z^{i+1}}{2}\right)^2\right] +$$
$$+ \frac{B}{2}\int d^2\vec{r}\left[(\partial_{xx} u_z^i)^2 + \left(\partial_{yy} u_z^i\right)^2\right] \qquad (2)$$

Where λ and μ are in plane elastic constants, Γ_b and Γ_s describes the interlayer breathing and shear stiffness, B is the bending rigidity of each layer, and d is the distance between layers.
The bending rigidity of a multilayer with N layers can be understood by analyzing the normal modes defined using the energy in eq. (2).

We focus on the low energy modes, described by smooth variations of the displacements between adjacent layers. A simple ansatz which is consistent with the continuum limit of isotropic slabs, which describes normal modes of eq. (2) is:

$$u_z^i(x) = h\sin(Gx), \qquad i = 1, \cdots, N$$
$$u_x^i(x) = \left(i - \frac{N+1}{2}\right)u_0\cos(Gx), \quad i = 1, \cdots, N \qquad (3)$$

with $G = G_x$, and h and u_0 are the amplitudes of out of plane and in plane displacements. The deformation described in eq. (3) does not require a change in the distance between layers, so that it does not depend on the out of plane elastic stiffness, Γ_b. The insertion of eq. (3) into eq. (2) leads to two modes for each value of G, depending on the relative sign between h and u_0. We consider the mode of lowest energy, $\omega_{fl}(G)$, whose dispersion is:

$$M\omega_{fl}^2(G)/(A/2) = \frac{1}{2}\left[\frac{N^3-N}{12}(\lambda+2\mu)G^2 + NBG^4 + \frac{(N-1)\Gamma_s}{d^2} + (N-1)\Gamma_s G^2\right] -$$
$$-\sqrt{\frac{1}{4}\left[\frac{N^3-N}{12}(\lambda+2\mu)G^2 - NBG^4 + \frac{(N-1)\Gamma_s}{d^2} - (N-1)\Gamma_s G^2\right]^2 + (N-1)^2\frac{\Gamma_s^2 G^2}{d^2}} \qquad (4)$$

Where A is the area of the unit cell, $M = 2 \times N \times M_C$ is the mass of the unit cell, and M_C is the mass of the carbon atom. Expanding eq. (4), we obtain:

$$M\omega_{fl}^2(G) \approx \begin{cases} \frac{N^3-N}{12}(\lambda+2\mu)G^4 d^2, & G^2 \ll \frac{12(N-1)\Gamma_s}{(N^3-N)d^2(\lambda+2\mu)} \\ N \times BG^4, & G^2 \gg \frac{12(N-1)\Gamma_s}{(N^3-N)d^2(\lambda+2\mu)} \end{cases} \qquad (5)$$

Which allows us to define an effective bending rigidity:

$$B_{eff}(G) \approx \begin{cases} \frac{N^3-N}{12}(\lambda+2\mu)d^2, & G^2 \ll \frac{12(N-1)\Gamma_s}{(N^3-N)d^2(\lambda+2\mu)} \\ N \times B, & G^2 \gg \frac{12(N-1)\Gamma_s}{(N^3-N)d^2(\lambda+2\mu)} \end{cases} \quad (6)$$

where the wavelength is $\ell = (2\pi)/G$. At long wavelengths, the effective bending rigidity scales as the cube of the number of layers, and it is determined by the in plane bulk modulus, in agreement with the general theory of elastic slabs (9). At short wavelengths, the bending rigidity scales like the number of layers, and it is proportional to the rigidity of a single layer. The crossover is determined by the shear stiffness.

We can use the paradigmatic case of graphene to gain some insight on the values of this crossover length. The elastic constants of single layer graphene are:

$$\begin{aligned} \lambda &\approx 2 \text{ eV Å}^{-2} \\ \mu &\approx 10 \text{ eV Å}^{-2} \\ B &\approx 1 \text{ eV} \end{aligned} \quad (7)$$

And the interlayer distance is $d \approx 3.5$ Å. The parameter Γ_s determines the shear mode of graphite and multilayered samples,

$$\omega_{sh}^2 = \frac{\Gamma_s A}{4M_C d^2} \quad (8)$$

where $A = (3\sqrt{3}a^2)/2$ is the area of the unit cell of graphene, and $a \approx 1.4$ Å is the distance between neighboring carbon atoms.

The frequency of the shear mode in bilayer graphene has been calculated and measured in Ref. (11), and a related calculation for multiwalled nanotubes can be found in Ref. (12). The Raman frequency of the interlayer shear mode in multilayered samples obtained in Ref. (11) depends on the number of layers, N. It ranges between $\omega_{fl} \approx c \times 31 \text{ cm}^{-1}$ for a bilayer and $\omega_{fl} \approx c \times 44 \text{ cm}^{-1}$ for graphite, where c is the velocity of light. The dependence of the shear frequency on the number of layers implies the existence of couplings between layers that are not nearest neighbors, not included in eq. (2). Neglecting this effect, we obtain the value of Γ_s, and a crossover length, ℓ^* between the two regimes described in eq. (6):

$$\Gamma_s \approx 17 \text{ meV Å}^{-2}$$

$$\ell^*(N) = \sqrt{\frac{4\pi^2(N^3-N)d^2(\lambda+2\mu)}{12(N-1)\Gamma_s}} \quad (9)$$

This length increases as the bulk modulus of a monolayer increases, and it decreases as the interlayer shear stiffness increases. For thick multilayers, we find $\lim_{N\to\infty} \ell^*(N) \approx 240 \times N \text{ nm}$, which is more than two orders of magnitude larger than the width of the stack, $W = (N-1) \times d$. For a graphene bilayer, the length is $\ell^* \approx 55$ nm.

In very rigid monolayers, the sliding of the monolayers is favored, and the bending rigidity of the stack is determined by the bending rigidity of each layer. On the other hand, in isotropic systems $\Gamma_s \sim \lambda + 2\mu$, and $\ell^* \sim N \times d$. The bending rigidity is independent of the bending rigidity of the individual layers for length scales larger than the width of the slab.

It is interesting to note that in twisted bilayers with a small twist angle most atoms in different layers are not in registry, and the interlayer shear is very small or it even vanishes, when the resulting Moiré pattern is incommensurate with the atomic lattices (12). Then, the bending rigidity of a small angle twisted graphene bilayer is always twice the bending rigidity of each layer.

References

1. B. Sachs, T. O. Wehling, M. I. Katsnelson, A. I. Lichtenstein, Adhesion and electronic structure of graphene on hexagonal boron nitride substrates. *Phys. Rev. B* 84, 195414 (2011).
2. Y. Y. Gan, H. J. Zhao, Chirality and vacancy effect on phonon dispersion of MoS2 with strain. *Phys. Lett. A* 380, 745-752 (2016).
3. S. K. Singh, M. Neek-Amal, S. Costamagna, F. M. Peeters, Thermomechanical properties of a single hexagonal boron nitride sheet. *Phys. Rev. B* 87, 184106 (2013).
4. K. Lai, W. B. Zhang, F. Zhou, F. Zeng, B. Y. Tang, Bending rigidity of transition metal dichalcogenide monolayers from first-principles. *J. Phys. D: Appl. Phys.* 49, 185301 (2016).
5. J. D. Paulsen, E. Hohlfeld, H. King, J. S. Huang, Z. L. Qiu, T. P. Russell, N. Menon, D. Vella, B. Davidovitch, Curvature-induced stiffness and the spatial variation of wavelength in wrinkled sheets. *Proc. Natl. Acad. Sci. U.S.A.* 113, 1144-1149 (2016).
6. N. Bowden, S. Brittain, A. G. Evans, J. W. Hutchinson, G. M. Whitesides, Spontaneous formation of ordered structures in thin films of metals supported on an elastomeric polymer. *Nature* 393, 146-149 (1998).
7. Q. Zhang, T. A. Witten, Microscopic wrinkles on supported surfactant monolayers. *Phys. Rev. E* 76, 041608 (2007).
8. E. Cerda, L. Mahadevan, Geometry and physics of wrinkling. *Phys. Rev. Lett.* 90, 074302 (2003).
9. L. Landau, E. Lifshitz, "Course of Theoretical Physics: Theory of Elasticity" (Pergamon Press, Oxford, 1959).
10. P. L. de Andres, F. Guinea, M. I. Katsnelson, Bending modes, anharmonic effects, and thermal expansion coefficient in single-layer and multilayer graphene. *Phys. Rev. B* 86, 144103 (2012).
11. P. H. Tan, W. P. Han, W. J. Zhao, Z. H. Wu, K. Chang, H. Wang, Y. F. Wang, N. Bonini, N. Marzari, N. Pugno, G. Savini, A. Lombardo, A. C. Ferrari, The shear mode of multilayer graphene. *Nat. Mater.* 11, 294-300 (2012).
12. A. N. Kolmogorov, V. H. Crespi, Smoothest bearings: Interlayer sliding in multiwalled carbon nanotubes. *Phys. Rev. Lett.* 85, 4727-4730 (2000).
